# Gene expression predicts dormant metastatic breast cancer cell phenotype

**DOI:** 10.1186/s13058-022-01503-5

**Published:** 2022-01-29

**Authors:** Qihao Ren, Weng Hua Khoo, Alexander P. Corr, Tri Giang Phan, Peter I. Croucher, Sheila A. Stewart

**Affiliations:** 1 Department of Cell Biology and Physiology, Washington University School of Medicine, 660 South Euclid Avenue, Campus, Box 8228, St. Louis, MO 63110 USA; 2 Garvan Institute of Medical Research, Sydney, NSW Australia; 3 St Vincent’s Clinical School, Faculty of Medicine and Healthy, UNSW Sydney, Sydney, NSW Australia; 4 Department of Medicine, Washington University School of Medicine, St. Louis, MO 63110 USA; 5 Siteman Cancer Center, Washington University School of Medicine, St. Louis, MO 63110 USA; 6 ICCE Institute, Washington University School of Medicine, St. Louis, MO 63110 USA

**Keywords:** Dormancy, scRNA-seq, Biomarkers, Breast cancer, Disseminated tumor cell

## Abstract

**Background:**

Breast cancer can recur months to decades after an initial diagnosis and treatment. The mechanisms that control tumor cell dormancy remain poorly understood, making it difficult to predict which patients will recur and thus benefit from more rigorous screening and treatments. Unfortunately, the extreme rarity of dormant DTCs has been a major obstacle to their study.

**Methods:**

To overcome this challenge, we developed an efficient system to isolate and study rare dormant breast cancer cells from metastatic organs including bones, which represent a major site of metastasis. After isolation of cells from the long bones, we used single cell RNA-sequencing (scRNA-seq) to profile proliferative and dormant PyMT-Bo1 breast cancer cells. We also compared this signature to dormant versus proliferative tumor cells isolated from the lungs. Finally, we compared our dormant signature to human datasets.

**Results:**

We identified a group of genes including *Cfh, Gas6, Mme* and *Ogn* that were highly expressed in dormant breast cancer cells present in the bone and lung. Expression of these genes had no impact on dormancy in murine models, but their expression correlated with disease-free survival in primary human breast cancer tumors, suggesting that these genes have predictive value in determining which patients are likely to recur.

**Conclusions:**

Dormant breast cancer cells exhibit a distinct gene expression signature regardless of metastatic site. Genes enriched in dormant breast cancer cells correlate with recurrence-free survival in breast cancer patients.

**Supplementary Information:**

The online version contains supplementary material available at 10.1186/s13058-022-01503-5.

## Background

Breast cancer is the most common cancer diagnosed among women worldwide and is the second leading cause of death [1]. Although most breast cancer patients are diagnosed at an early stage and successfully treated, ~ 20–30% experience cancer recurrence months to years later. Unfortunately, late breast cancer recurrences (> 5 years) account for most of the deaths among this patient population [2]. These findings raise important questions including, (1) when do disseminated tumor cells (DTCs) leave the primary site and (2) what controls whether they go on to form deadly metastatic disease? Studies in the MMTV-Her2-CFP genetically engineered mouse model (GEMM) demonstrated that DTCs could be reliably found in multiple organs, including lung and bone, before primary tumor masses were detectable [3]. These early DTCs were dormant and stained negative for proliferation markers, but eventually gave rise to metastases. These observations suggest that dormant DTCs metastasize to the bone and lung early. Similarly, DTCs have been found in the bone marrow of patients with local disease where their presence is a prognosis marker for recurrence [4, 5]. Thus, in both patients and murine models, tumor cells have the capacity to metastasize early and remain dormant for months to decades. Uncovering the mechanisms that govern entrance and emergence from dormancy could have an important impact on not only our understanding of dormancy but also treatment choices and patient outcomes.

Unfortunately, the extreme rarity of DTCs in a patient’s bone marrow has been a major obstacle to exploring the mechanisms that regulate DTC dormancy and reactivation. Taking advantage of single-cell sequencing technologies, researchers have now profiled rare dormant cancer cells and have identified critical intrinsic signaling pathways that govern dormancy in myeloma and prostate cancer [6, 7]. Additionally, several studies successfully identified key niche components within the bone that maintain cancer cells in a dormant state [8–12], and changes in these niches that can lead to the outgrowth of dormant cancer cells [9, 11, 13–17].

In this study, we developed an efficient system for isolating rare dormant breast cancer cells in experimental metastases models and performed scRNA-seq comparing gene expression in dormant vs proliferative breast cancer cells. Through this approach, we identified a group of dormancy-related genes. The expression of dormancy-related genes identified in our analyses was consistently observed among different breast cancer dormancy models and correlated with breast cancer progression in patients. Our studies suggest that the gene expression signature we identified in disseminated dormant breast cancer cells exists in the primary site and can be utilized to predict which patients are more likely to experience a breast cancer recurrence. Further, these finding could lead to the development of novel treatments targeting dormant breast cancer cells in those patients more likely to experience a recurrence of their disease.

## Methods

### Cell lines and cell culture

The PyMT-Bo1 breast cancer cell line (C57BL6 background) was obtained from Dr. Katherine Weilbaecher's laboratory. The D2A1 and D2.0R breast cancer cell lines (BALB/c background) were gifted by Dr. Sandra McAllister. 293T cells were obtained from Dr. Robert Weinberg. All cell lines were cultured in DMEM high glucose with 10% heat-inactivated FBS and 1% Pen-Strep. All cells were cultured in 5% CO2 and 37 °C. Trypsin-EDTA (0.05%) was used for passaging and harvesting.

### Lentivirus production and plasmid vectors

Lentivirus production and transduction were carried out according to Addgene protocol (https://www.addgene.org/protocols/lentivirus-production/). The EGFP gene in the FUGW plasmid (Addgene ##14883) was replaced by H2B-mApple, Thy1.1 and Luc2-EGFP separately to label PyMT-Bo1 cells. Transduced PyMT-Bo1 cells were purified by FACS sorting. Lenti-luciferase-P2A-Neo (Addgene #105621) was used to express the luciferase gene in D2A1 and D2.0R cells because of the known immunogenicity of EGFP in BALB/c mice [18]. Transduced D2A1 and D2.0R cells were selected for in 500 µg/ml G418 (Gibco). The LentiCRISPR v2 plasmid (Addgene #52961) was used to knock out dormancy-related genes in D2.0R cells with gRNA sequence targeting selected dormancy genes (Additional file 4: Supplementary Table S3). For overexpression in PyMT-Bo1 cells, the Cas9 gene in pLenti-Cas9-P2A-Puro plasmid (Addgene #110837) was replaced with cDNAs by Gibson assembly. Knockout and overexpression cells were selected by 2 µg/ml puromycin (Sigma).

### Mice

All mice used in this study were 8–10 weeks old female mice purchased from the Jackson Laboratory. Wild-type C57BL6/J mice (Stock#: 000664) were used for FACS isolation of PyMT-Bo1 cells. Dormancy-gene overexpressed PyMT-Bo1 cells were injected into B6(Cg)-Tyr^c−2 J^/J albino mice (Stock#: 000058) to compare in vivo metastatic ability. BALB/cJ (Stock#: 000651) mice were used for experiments involving D2A1 and D2.0R cells. All mice were directly purchased from the Jackson Laboratory and housed for at least one week before being used for experiments. All animal experiments were in accordance with Washington University in St. Louis's Studies Committee and Institutional Animal Care and Use Committee (IACUC).

### Membrane staining and injection of breast cancer cells

Vybrant™ DiD Cell-Labeling Solution (Invitrogen) was used to stain adherent breast cancer cells directly in petri dish. Specifically, DiD solution was added 5 µl/ml into complete culture medium and mixed well before adding to adherent breast cancer cells. Cells were stained for 8 h and replaced with fresh complete culture medium 1d before injection to remove the excess dye. For intravenous (IV) injection, 500,000 breast cancer cells are injected into restrained mice without anesthesia. Ketamine/Xylazine solution was used for anesthesia during intracardiac (IC) and intra-tibial (IT) injection. 500,000 breast cancer cells in 50 µl PBS are injected into the left ventricle of heart for IC injection, while 5000 breast cancer cells in 10ul PBS are injected directly into tibia for IT injection. 29 G insulin syringes were used for IV, IC and IT injections.

### Fluorescence imaging of tumor cells in the bone

Mice were perfused with Heparin (Alfa Aesar) solution followed by 4% PFA (Microscopy Sciences) before harvesting tissues for fluorescence imaging. Bones were fixed in 4% PFA overnight. Zeiss LSM 880 Airyscan Two-Photon Confocal Microscope was used to image PyMT-Bo1 metastatic lesions in the intact bones. To image DiD + cells in the bone, harvested bones were decalcified in 14% EDTA pH = 7.2 for 3 days before cryo-sectioning (10 µm sections). Samples were mounted with Fluoroshield containing DAPI (Sigma) to preserve the DiD fluorescence and imaged by Nikon Eclipse Ti-E microscope.

### Breast cancer cell isolation from bone and lung

IC injected mice were euthanized according to IACUC guidelines. The method for tumor cell isolation from bone has been described in the RESULTs section. Briefly, muscle and connective tissue are removed from the femur and tibia. Bones were then ground with a mortar and pestle in 2 mg/ml Collagenase Type I (Sigma) dissolved in DMEM/F12. Ground bone pieces were cut into even smaller pieces with a scissor and transferred to bottles in a water bath for collagenase digestion at 37 °C. After a 25-min digestion, supernatant (1) was collected on ice and fresh collagenase digestion solution was added back for further digestion. After two more rounds of 25 min digestion, supernatant (2) and (3) were collected again on ice and the remaining bone pieces were washed with FACS buffer 3 times to collect all the released cells. Supernatant (1), (2), (3) and FACS buffer (0.5% BSA in PBS with 2 nM EDTA) collections were combined and filtered through 40 µm cell strainer to obtain a single cell suspension. Red blood cells were lysed using RBS lysis buffer (BioLegend) for 5 min on ice. For lung metastases, lungs were collected and cut into 1–2 mm^3^ pieces for digestion. Lung tissues were digested in 2 mg/ml collagenase solution for 45 min. Digested tissues were passed through a 40 µm cell strainer and subjected to RBC lysis as above.

### Collection of dormant and proliferative PyMT-Bo1 cells for scRNA-seq

Single cell suspensions from digested bones and lungs were first enriched for PyMT-Bo1 tumor cells using Thy1.1 MACS beads (Miltenyi Biotec) according to the manufacturer protocol. After MACS enrichment, cells were blocked with 1:200 anti-mouse CD16/32 (2.4G2) for 10 min and then stained with 1:200 anti-mouse CD45 (30-F11, PacBlue) and 1:200 anti-mouse Thy1.1 (OX-7, PE-Cy7) in FACS buffer (0.5% BSA in PBS with 2 mM EDTA). 0.1 µg/ml DAPI (Sigma) was added to the sample 5 min before FACS analysis to stain for dead cells. PyMT-Bo1 cells were sorted into 96-well plates containing 2 µl of 10 × Lysis buffer (Clontech) with 5% RNase inhibitor (Clontech), 1 cell per well. Plates were then sealed, spun and stored in − 80 °C before proceeding to scRNA-seq. A Sony SY3200 cell sorter was used for single cell sorting.

### scRNA-seq and analysis

Single‐cell cDNA was generated using the SMARTer Ultra Low RNA Kit v4 (Takara) with modifications as previously described [6]. Briefly, 1:2,500,000 dilution of ERCC spike‐in controls (Ambion) was incorporated during first‐strand cDNA synthesis and subsequent steps were performed according to the manufacturer's instructions at half‐reaction volumes. cDNA amplification was performed at 18 cycles and its quality was assessed using the Bioanalyzer HS DNA chip (Agilent Technologies) according to the manufacturer's instructions. Sequencing libraries were generated using 1 ng of input material using the Nextera XT Kit (Illumina) according to the manufacturer's protocol at half-reaction volumes. Libraries were pooled and paired-end sequenced (125‐bp reads) across 2 lanes on Illumina HiSeq2500 on a high‐throughput mode.

### scRNA-seq data preprocessing and normalization

Illumina sequence adapters were trimmed, and reads were aligned to a modified version of the GRCm38/mm10 mouse genome (supplemented with ERCC, mApple, eGFP, luciferase, PyMT oncogene sequences) using the STAR aligner [19]. Summarized gene transcript counts and TPMs were generated using RSEM [20]. All subsequent normalization and differential expression analyses were performed using the BASiCS package according to methods previously described. Visualization of the differentially expressed genes (DEG) between the dormant and reactivated PyMT cells was performed by rescaling the gene expression in the 10% and 90% quantile and removing genes which failed the rescaling process. The Heatmap was generated using the ComplexHeatmap package in R [21].

### Low-input qPCR for sorted cells

Tumor cells were sorted 20 cells per well using BD FACSAria II Cell Sorter. CellsDirect™ One-Step qRT-PCR Kit (Invitrogen) was used to isolate RNA, generate cDNA and pre-Amplification (15 cycles) of sorted tumor cells. Probes for pre-Amp and qPCR were purchased from IDT (Additional file 3: Supplementary Table S2). qPCR was carried out using PrimeTime™ Gene Expression Master Mix (IDT) and the Bio-Rad CFX96 Touch Real-Time PCR Detection System.

### Bioluminescence imaging

All bioluminescence imaging experiments were performed on a Xenogen IVIS50. For in vivo experiments, mice were injected with 150 mg/kg D-Luciferin (Gold Biotechnology) in PBS and imaged under anesthesia (2% isoflurane vaporized in O_2_) 10 min later. Depending on the tumor burden, exposure time varied from 1 s to 3 min. For in vitro experiments, cells or bone pieces were incubated in 0.15 mg/ml D-Luciferin in DMEM/F12 medium for 10 min at 37 °C before BLI.

### Statistics analysis

All statistical analyses were carried out using Graphpad Prism. Numerical data are expressed as mean + / − SEM. Specific statistic analysis approaches were described in each figure legend. Kaplan–Meier Plots were generated online from Kaplan–Meier Plotter (https://kmplot.com/analysis/). [22]

## Results

### Development of a robust system to isolate rare DTCs from bone

**Fig. 1:**
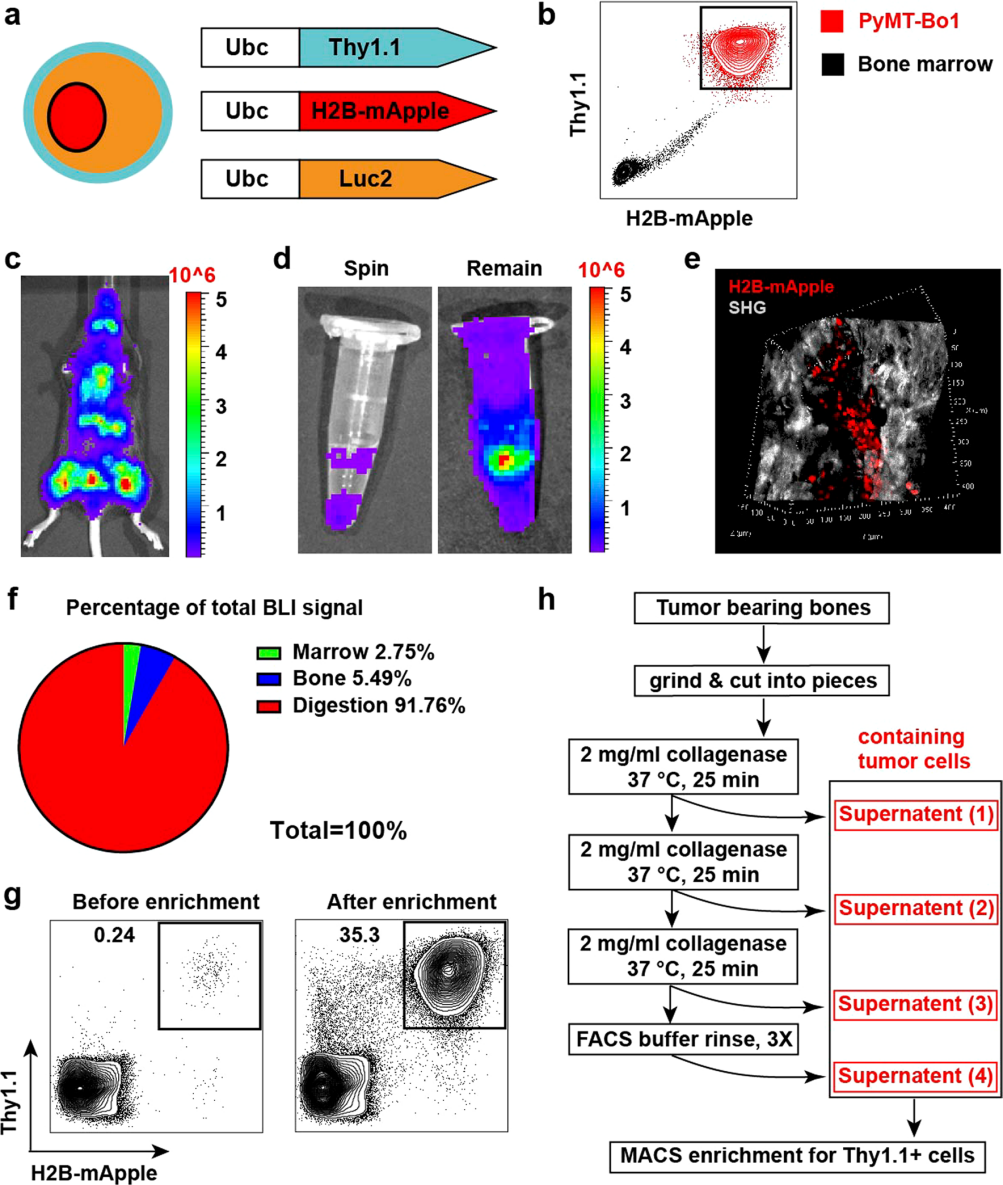
Development of an efficient tumor cell isolation system for DTCs in the bone. **a** PyMT-Bo1 labeling strategy using Thy1.1, H2B-mApple and Luc2. **b** Flow cytometry analysis comparing tri-labeled PyMT-Bo1 tumor cells admixed with murine wildtype bone marrow cells. **c** BLI image of WT C57BL6/J mice 9 days after IC injection of 500,000 PyMT-Bo1 cells. Scale represents photon flux (photons/sec/cm^2^/sr). **d** Representative ex vivo BLI image comparing PyMT-Bo1 cells spun from the bone marrow versus those that remain attached to the bone. Scale represents photon flux (photons/sec/cm^2^/sr). **e** Two-photon microscopy image of tumor-bearing femur. 3D reconstruction size: 425 × 425 × 168 µm^3^ (Red: mApple + tumor cell nucleus; Gray: second hormonic generation of collagen fibers) **f** Summary of PyMT-Bo1 signal in different fractions of the bone after 3 rounds of collagenase digestion. **g** Flow cytometry analysis of PyMT-Bo1 enrichment before and after Thy1.1 MACS. **h** Schematic of final tumor cell isolation strategy from bone

In breast cancer patients, bone is the most common site of metastasis and DTCs have been found in the bones of patients with early-stage disease [23], suggesting the DTCs reside in the bone in a dormant state. To study dormant breast cancer cells disseminated to bone, we developed a tri-label system to facilitate their isolation (Fig. 1a). We expressed the firefly luciferase gene in PyMT-Bo1 breast cancer cells [24] to monitor their metastatic growth in vivo*.* Additionally, the H2B-mApple fusion gene was introduced to label the PyMT-Bo1 nucleus with bright red fluorescence. To further facilitate identification of our cells, we introduced a congenic cell surface marker *Thy1.1* into PyMT-Bo1 cells, which distinguished tumor cells from host cells in wild type C57BL6 mice that express *Thy1.2*. Strong promoters such as the CMV promoter are often subject to inactivation [25, 26]. Therefore, to ensure persistent expression *in* vivo, we chose the eukaryotic Ubc promoter to drive transgene expression. As a result, we found that our tri-labeled PyMT-Bo1 cells could be clearly distinguished from host cells. Using flow cytometry, we found a distinct mApple^+^Thy1^+^ population when tri-labeled PyMT-Bo1 tumor cells were mixed with bone marrow cells (Fig. 1b). Importantly, the addition of the Thy1.1 surface marker did not affect the metastatic characteristics of PyMT-Bo1 cells as evidenced by their ability to grow as robustly as PyMT-Bo1 cells that do not express *Thy1.1* (Additional file 1: Fig. S1a–d).

Having developed a labeling approach to identify PyMT-Bo1 cells, we next aimed to establish a robust approach to isolate them from the bone after intracardiac (IC) injection (Fig. 1c). However, unlike bone marrow cells or leukemic cancer cells, we found that flushing the bone marrow or centrifugation of the bone to remove the marrow was insufficient to separate tumor cells from mouse long bones (Fig. 1d). Indeed, whole-mount two-photon imaging revealed a close association between PyMT-Bo1 cells and the bone matrix (Fig. 1e). In line with Welte et al [27], routine enzymatic digestion only released a small fraction of the tumor cells from the bone and left the majority of tumor signal on the remaining bone pieces. To overcome this challenge, and balance isolation with viability, we adapted an osteocyte isolation approach [28]. Tumor-bearing bones were cut and ground into small pieces in 2 mg/ml collagenase type I and further subjected to three rounds of enzymatic digestion. Using this approach, we successfully released most PyMT-Bo1 cells from the bone as evidenced by the small amount of luciferase signal remaining on the enzymatically treated, smashed bone pieces (Fig. 1f). Extended digestion time recovered a few additional tumor cells as evidenced by increased luciferase activity (Additional file 1: Fig. S2a, b). Finally, to minimize sorting time and increase the viability of our cells, we optimized the isolation of the released tumor cells by taking advantage of the exogenous congenic cell surface marker Thy1.1. Released PyMT-Bo1 cells were enriched from bone marrow cells by using Magnetic-activated cell sorting (MACS), which resulted in a more than 100-fold enrichment (Fig. 1g). Importantly, we found that this approach resulted in the isolation of viable cells suitable for downstream analyses such as single cell RNA-sequencing (scRNA-seq). In summary, we developed an efficient system to isolate rare breast cancer cells from the bone microenvironment (Fig. 1h).

### Isolation of dormant breast cancer cells from bone

With an optimized system for recovering dormant breast cancer cells from the bone in hand, we next used fluorescent activated cytometric sorting (FACS) to sort dormant and proliferative PyMT-Bo1 cells from the bones of mice. To track dormant versus proliferative cells, we first compared two popular membrane dyes that have been used to track cell proliferation. For these studies, we compared the Vybrant DiD (DiD) membrane dye or CellTrace Far Red (CTFR, similar to CFSE), both of which are diluted ~ 50% after each cell division. To assess the durability of each dye, we lethally radiated murine fibroblasts (30 Gy) to halt their proliferation and labeled them with DiD or CTFR. We found that the DiD membrane dye was superior for long-term cell tracking because irradiated fibroblasts maintained the initial level of DiD staining over seven days while CTFR fluorescence was lost significantly over the same time course (Additional file 1: Fig. S3a). Thus, we choose to use DiD for subsequent studies as it would allow us to identify proliferating versus nonproliferating label-retaining cells (LRCs) in vivo over time.

**Fig. 2:**
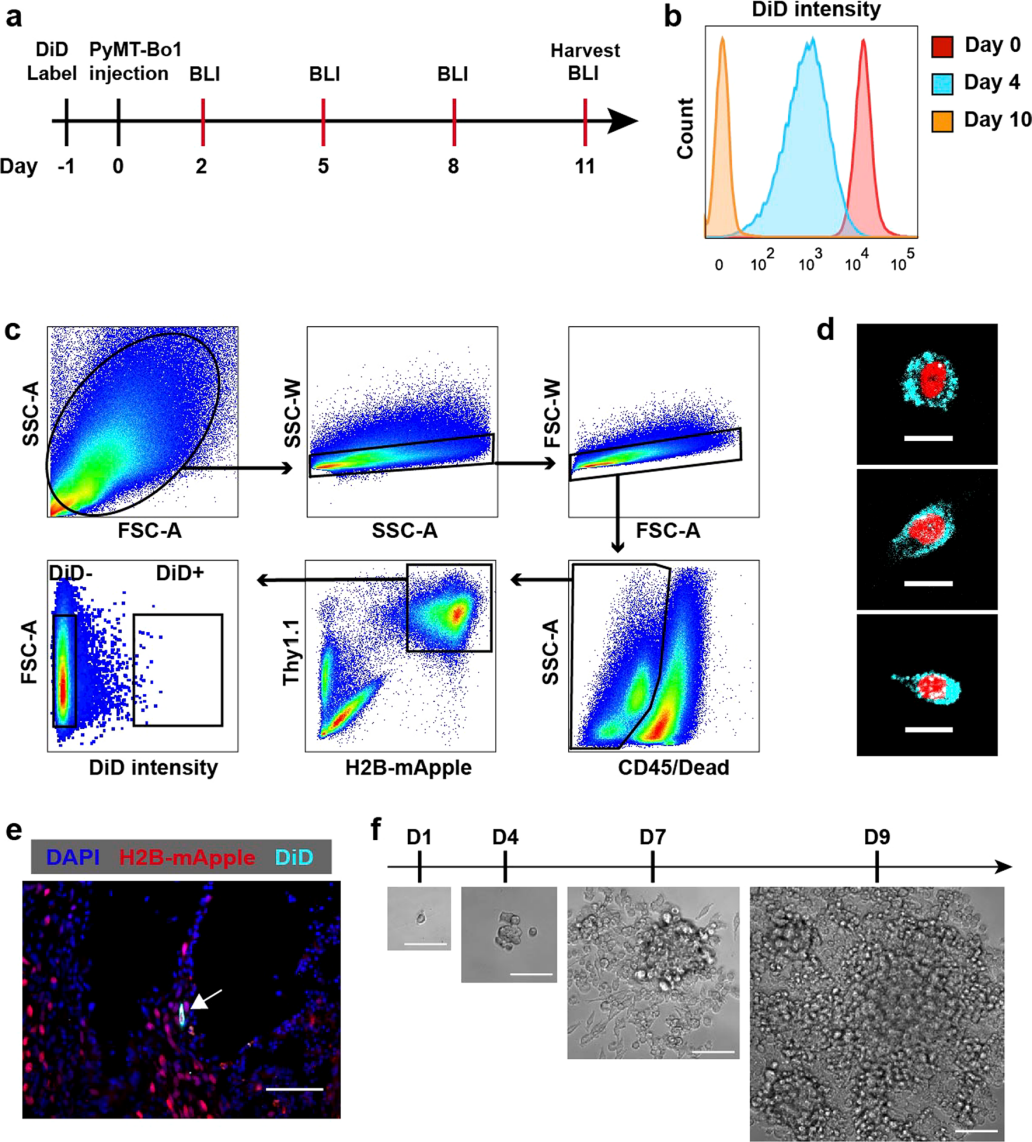
Isolation of dormant PyMT-Bo1 cells from bone for scRNA-seq. **a** Schematic of the experimental timeline for dormant PyMT-Bo1 isolation. **b** Dilution of DiD membrane dye fluorescence following PyMT-Bo1 cell division in vitro. **c** Gating strategy for FACS sorting of DiD + and DiD- PyMT-Bo1 cells for scRNA-seq. **d** Fluorescence image of sorted DiD + PyMT-Bo1 cells. (Red: mApple + tumor nucleus; Cyan: DiD fluorescence; Scale bar = 10 µm) **e** Fluorescence image of dormant DiD + PyMT-Bo1 cells in situ in the bone. (Red: mApple + tumor nucleus; Cyan: DiD fluorescence; Blue: DAPI; White arrow points to a DiD + tumor cell, Scale bar = 80 µm), **f** Tracking sorted DiD + PyMT-Bo1 cell proliferation in vitro over time as indicated on the timeline. (Scale bar = 100 µm)

For breast cancer, DTCs are found in upwards of 50% of patients with primary disease, yet most these patients do not go on to experience a recurrence [29]. Thus, our goal was to isolate and characterize DTCs from bones to determine what controls their ability to proliferate in distal organs. To obtain LRCs from the bone, DiD-stained tri-labeled PyMT-Bo1 cells were introduced into mice by IC injection, which can deliver tumor cells to the bone and other metastatic sites. Following IC delivery of cells, we followed tumor cell growth by bioluminescence imaging (BLI) and found that cells grew aggressively and maintained an exponential growth rate (Additional file 1: Fig. S3b). Cells were harvested 11 days after IC delivery, when proliferative PyMT-Bo1 cells had lost all DiD fluorescence in vitro (Fig. 2a and 2b). PyMT-Bo1 cells were isolated from both femurs and tibias of 5 IC injected mice using the approach described above for FACS sorting. To increase our sorting efficiency, cells were stained for CD45 and DAPI to exclude hematopoietic cells and dead cells, respectively. Using this approach, we found that DiD + dormant PyMT-Bo1 cells were rare in the bone but could nonetheless be reliably detected using flow cytometry (Fig. 2c). Fluorescence microscopy confirmed that sorted DiD + mApple + tumor cells displayed high DiD fluorescence intensity (Fig. 2d). We also confirmed the presence of DiD + cells inside the bones of mice by fluorescence microscopy in situ (Fig. 2e). The presence of the dye retaining cells in vivo raised the possibility that they were dormant and could seed future metastatic lesions or that they were arrested and incapable of re-entering the cell cycle. To ensure that DiD + cells retained the ability to divide, we isolated them and plated them into complete culture medium where we found they efficiently re-entered the cell cycle and started to proliferate, indicating that DiD + PyMT-Bo1 cells are viable dormant breast cancer cells that retain the ability to reactivate and divide (Fig. 2f). Having established our labeling and isolation procedure, we next sorted single DiD + dormant and DiD-proliferative PyMT-Bo1 cells into 96-well plates for downstream scRNA-seq analysis.

### scRNA-Seq profiling of dormant vs proliferative breast cancer cells

**Fig. 3:**
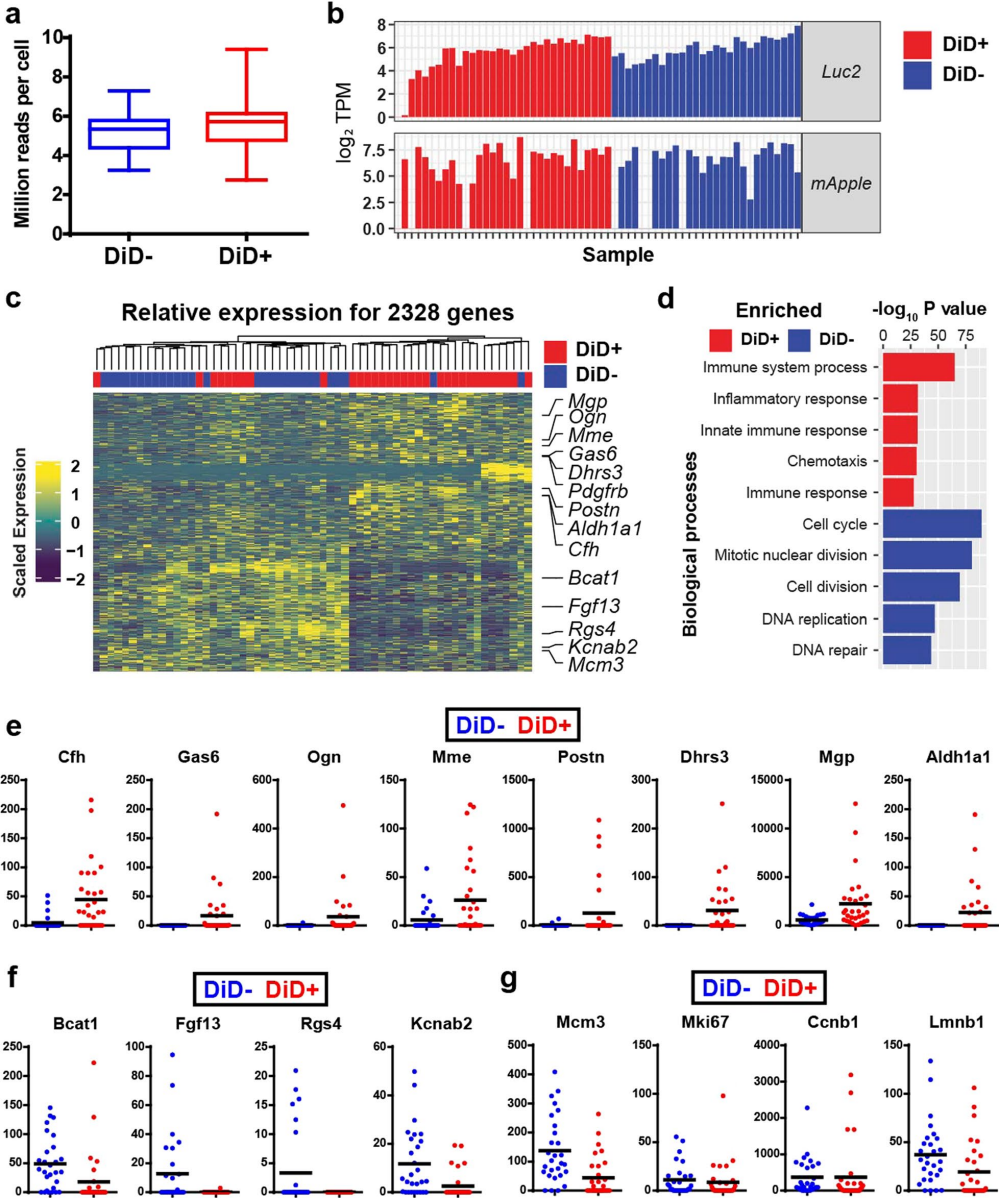
scRNA-seq analysis of dormant vs proliferative PyMT-Bo1 cells from bone. **a** Distribution of sequencing read coverage across single dormant (DiD +) vs proliferative (DiD-) PyMT-Bo1 cells. **b** Transcript profile of DiD- and DiD + single cells showing expression levels of luc2 and mApple. **c** Heatmap of 3673 differentially expressed genes in dormant (DiD +) versus proliferative (DiD-) cells. **d** Gene set enrichment analysis revealing biological processes enriched in dormant (DiD +) and proliferative (DiD-) PyMT-Bo1 cells, respectively. **e** Transcript profile (transcripts per million, TPM) on the Y axis of selected up-regulated genes in dormant DiD + and proliferative DiD- PyMT-Bo1 cells. **f** Transcript profile (transcripts per million, TPM) on the Y axis of top down-regulated genes in dormant DiD + and proliferative DiD- PyMT-Bo1 cells. **g** Transcript profile (transcripts per million, TPM) of selected cell cycle related genes in dormant DiD + and proliferative DiD- PyMT-Bo1 cells

Using our isolation approach, we successfully isolated 48 DiD- and 48 DiD + PyMT-Bo1 cells from the bones of mice. Among the 96 sorted cells, 28 DiD- and 32 DiD + PyMT-Bo1 cells passed quality control and were subjected to RNA sequencing. On average, we obtained ~ 5 million RNA-Seq reads per cell regardless of DiD status (Fig. 3a) and the vast majority of reads uniquely mapped to the mouse genome (Fig. S4a). Further, we found that external RNA Controls Consortium (ERCC) spike-ins during library preparation exhibited a strong correlation and equivalent mean read coverage per depth across all cells (Fig. S4b). In addition, expression of at least one transgene, including *mApple* and *Luc2*, were detected among all the cells and the average expression level was identical between DiD- and DiD + groups, while no *Ptprc* (*CD45*) expression was detected (Fig. 3b and data not shown). These data indicated that our single cell sequencing was performed on bona fide PyMT-Bo1 cells without any host cell contamination.

We next wanted to determine how the gene expression pattern changed in DiD + versus DiD- cells, so we carried out differential gene expression (DEG) analysis using BASiCS software. Our analysis revealed 3673 differentially expressed genes when comparing DiD + and DiD- cells, among which 2329 genes were up-regulated, and 1344 genes were down-regulated (Additional file 2: Supplementary Table S1) in dormant PyMT-Bo1 cells (Fig. 3c). As expected, gene ontology analysis of differentially expressed genes revealed that biological processes including cell cycle, cell division and DNA replication were enriched in DiD proliferative PyMT-Bo1 cells (Fig. 3d). In contrast, genes involved in immune system processes such as inflammatory responses and immune responses were enriched in dormant cells (Fig. 3d). Quantification results (transcript per million, TPM) of significantly enriched genes in DiD + (Fig. 3e) and DiD- (Fig. 3f) cells were plotted as were proliferation markers (Fig. 3g). Several differentially expressed genes in DiD + dormant cells have already been reported to associate with dormancy in prostate and multiple myeloma cancer cells, including *Cfh* [17], *Gas6* [16], *Nr2f1* [17, 30], *Bhlhe41* [31], *Irf7* [7], *Thbs1* [8] and *Aldh1a1* [32] (Fig. 3e and Additional file 1: Fig. S4c). This observation further suggested that sorted DiD + PyMT-Bo1 cells represent a bona fide dormant cancer cell population and suggests that there may be a common gene expression signature that distinguishes dormant cancer cells across tissue types.

### Dormant PyMT-Bo1 cells exist in the lung and bone, exhibiting similar gene expression signatures

**Fig. 4:**
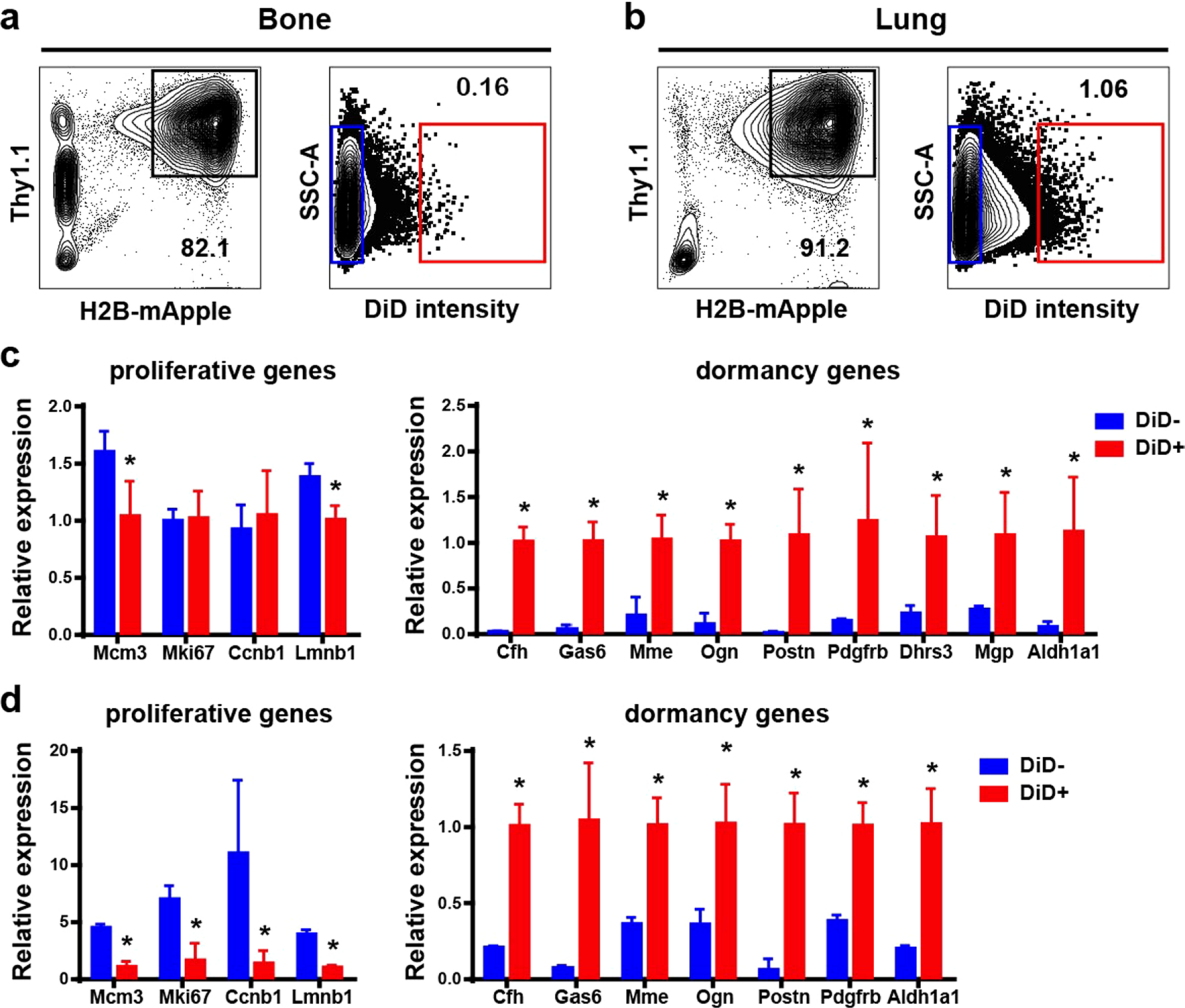
Dormant PyMT-Bo1 cells exhibit a similar gene expression signature in both lung and bone. **a**, **b** FACS sorting DiD + and DiD- PyMT-Bo1 cells from bone (**a**) and lung (**b**) for qPCR validation. **c**, **d** qRT-PCR results comparing proliferation and dormancy-related genes in dormant DiD + and proliferative DiD- PyMT-Bo1 cells (*n* = 4) sorted from bone (**c**) and lung (**d**). Significance was determined by unpaired t tests, *p ≤ 0.05

To determine if the expression changes obtained from DiD + PyMT-Bo1 cells in the bone were cell intrinsic or the result of seeding into the bone microenvironment, we next used a similar approach to isolate DiD + and DiD- cells from both the lungs and bones of mice and used qRT-PCR to compare the expression of selected genes. Because IC injection can deliver cancer cells to multiple metastatic sites, we isolated and analyzed PyMT-Bo1 cells from the bones (Fig. 4a) and lungs (Fig. 4b) of the same mice 11 days after tumor cell injection. Interestingly, dormant PyMT-Bo1 cells with high DiD fluorescence intensity were also detected in the lung, suggesting bone is not the only place to harbor dormant breast cancer cells. qRT-PCR from DiD + cells isolated from bone confirmed that our dormancy-related genes including *Cfh, Gas6, Mme, Ogn, Postn, Pdgfrb, Aldh1a1, Dhrs3* and *Mgp* that we had identified by scRNA-seq were expressed at much higher levels in DiD + compared to DiD- cells (Fig. 4c). According to our scRNA-seq analysis, many proliferation-associated genes were up-regulated in DiD- PyMT-Bo1 cells with moderate fold changes and significant *p*-values. Therefore, we chose a few widely used proliferation markers and compared their expression by qRT-PCR in isolated DiD- or DiD + cells. Indeed, our qPCR results fit well with our sequencing results, demonstrating that Mcm3 and Lmnb1 were increased in DiD low/negative cells from bone (Fig. 4c and Fig. 3g). Additionally, we detected similar expression levels of the *Luc2* transgene and no CD45 contamination in both DiD + and DiD- sorted PyMT-Bo1 cells (Additional file 1: Fig. S5). This finding further supports the accuracy of the scRNA-seq data. To our surprise, DiD + PyMT-Bo1 cells isolated from lungs exhibited the same gene expression signature as DiD + cells isolated from bones (Fig. 4d). Proliferation genes were expressed at much lower levels in lung DiD + PyMT-Bo1 cells consistently while *Cfh, Gas6, Mme, Ogn, Postn, Pdgfrb* and *Aldh1a1* were all increased in DiD + cells. These observations demonstrate a common gene signature that regulates breast cancer cell dormancy regardless of their seeding site.

### Dormancy gene expression signature is present in indolent D2.0R breast cancer cells

**Fig. 5:**
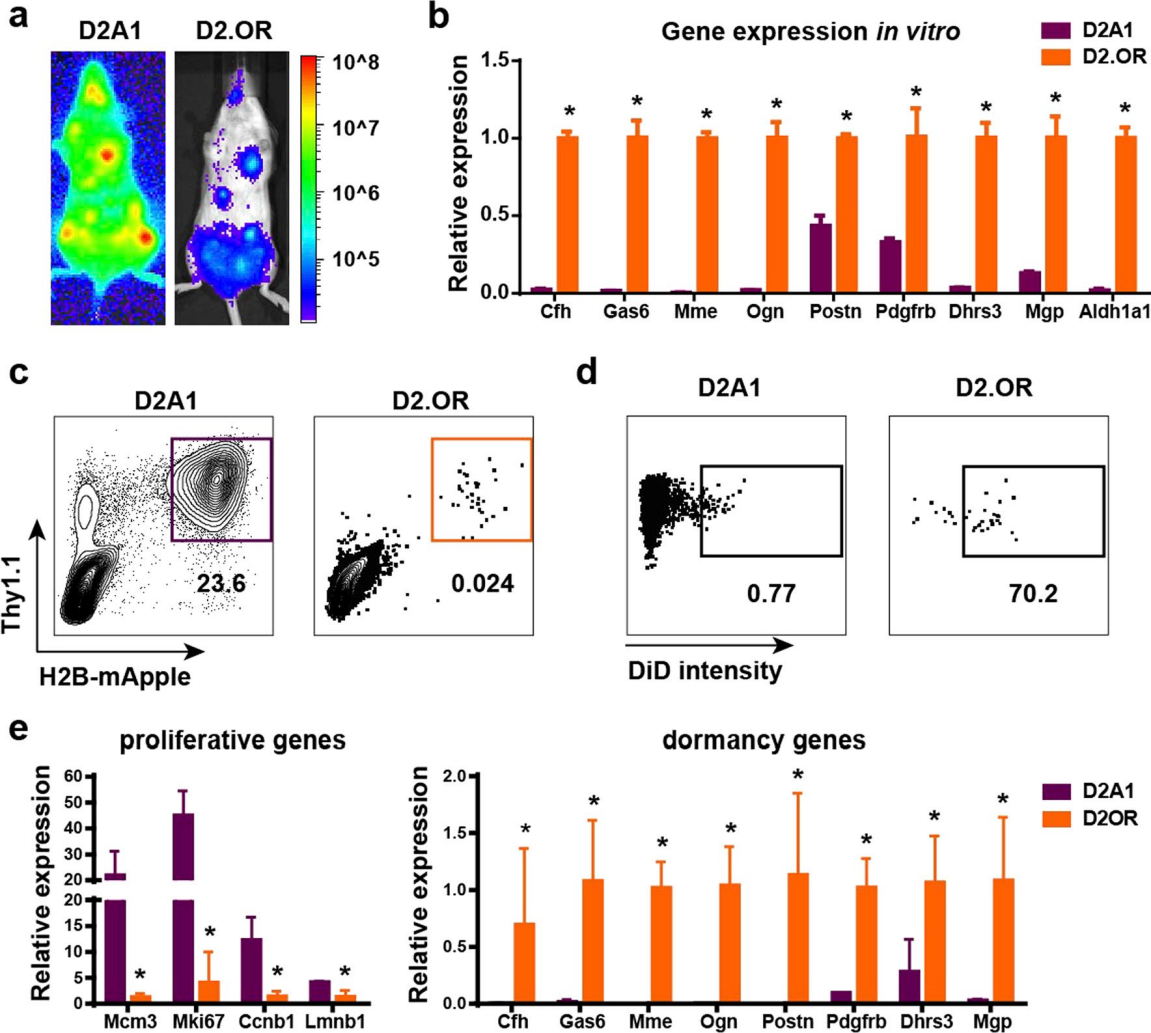
Dormancy signature in D2.0R breast cancer cells. **a** Whole body BLI of mice IC injected with D2A1 or D2.0R cells. **b** qRT-PCR comparing dormancy gene expression in D2A1 and D2OR cell lines in vitro (*n* = 3). **c** FACS sorting of D2A1 and D2.0R cells from lungs 2 weeks after IV injection. **d** DiD fluorescence intensity on sorted D2A1 and D2.0R cells from lung. **e** qRT-PCR results comparing proliferation and dormancy-related genes in sorted D2A1 and D2.0R cells (*n* = 3). Significance was determined by unpaired t tests, *p ≤ 0.05

To determine if our gene signature was broadly applicable to dormant breast cancer cells, we examined its expression in an alternative model. For this work, we utilized the D2A1 and D2.0R breast cancer cell lines that were both derived from the same spontaneous breast cancer model in BALB/c mice [33]. Although D2A1 and D2.0R proliferate at comparable rates in vitro (Additional file 1: Fig. S6a), they exhibit drastically different metastatic potential in vivo. In agreement with a previous study [34], we found that D2A1 cells grow aggressively in multiple organs, while D2.0R cells survive in multiple organs but rarely show any metastatic growth (Fig. 5a). We first compared the expression levels of our dormancy-related genes in D2A1 vs D2.0R cells in vitro and found that our dormancy-related genes were expressed at much higher levels in D2.0R cells compared to D2A1cells (Fig. 5b), suggesting that the genes we identified in DiD + cells do not control proliferation of dormant breast cancer cells but rather function as dormancy markers for cancer cells. Further, this finding suggested that our signature might be broadly applicable across cell lines. To determine if expression was maintained in vivo, tri-labeled (Luc2, H2B-mApple and Thy1.1) D2A1 and D2.0R cells were introduced into mice. When D2A1 and D2.0R cells were IC injected into BALB/c mice, we had difficulty recovering enough D2.0R cells from the bone for analysis despite our efficient isolation system (Additional file 1: Fig. S6b). However, we successfully isolated sufficient cell numbers of D2.0R DiD + cells that were present in the bone as single cells (Additional file 1: Fig. S6c) for qRT-PCR analysis from lung after an intravenous (IV) injection and bone after intra-tibial (IT) injection (Fig. 5c and Additional file 1: Fig. S6d), which allows greater numbers of cells to be delivered to the bone. Importantly, the D2.0R cells obtained from the lung maintained high levels of DiD fluorescence for two weeks, while most D2A1 cells lost nearly all DiD fluorescence and continued to grow (Fig. 5d), indicating that D2.0R cells can remain dormant in vivo for extended periods of time. qRT-PCR analysis of dormant D2.0R cells isolated from the lungs revealed that dormancy-related genes including *Cfh, Gas6, Mme, Ogn, Postn, Pdgfrb, Dhrs3* and *Mgp* were highly expressed in D2.0R cells, while proliferation genes including *Mcm3, Ccnb1, Mki67* and *Lmnb1* were expressed at higher levels in metastatic D2A1 cells isolated from lungs (Fig. 5e) confirming our earlier findings with PyMT-Bo1 cells. A similar expression pattern was also observed in D2.0R and D2A1 cells isolated from the bone after IT injection (Additional file 1: Fig. S6e).

### Interrogation of dormancy-related genes

**Fig. 6:**
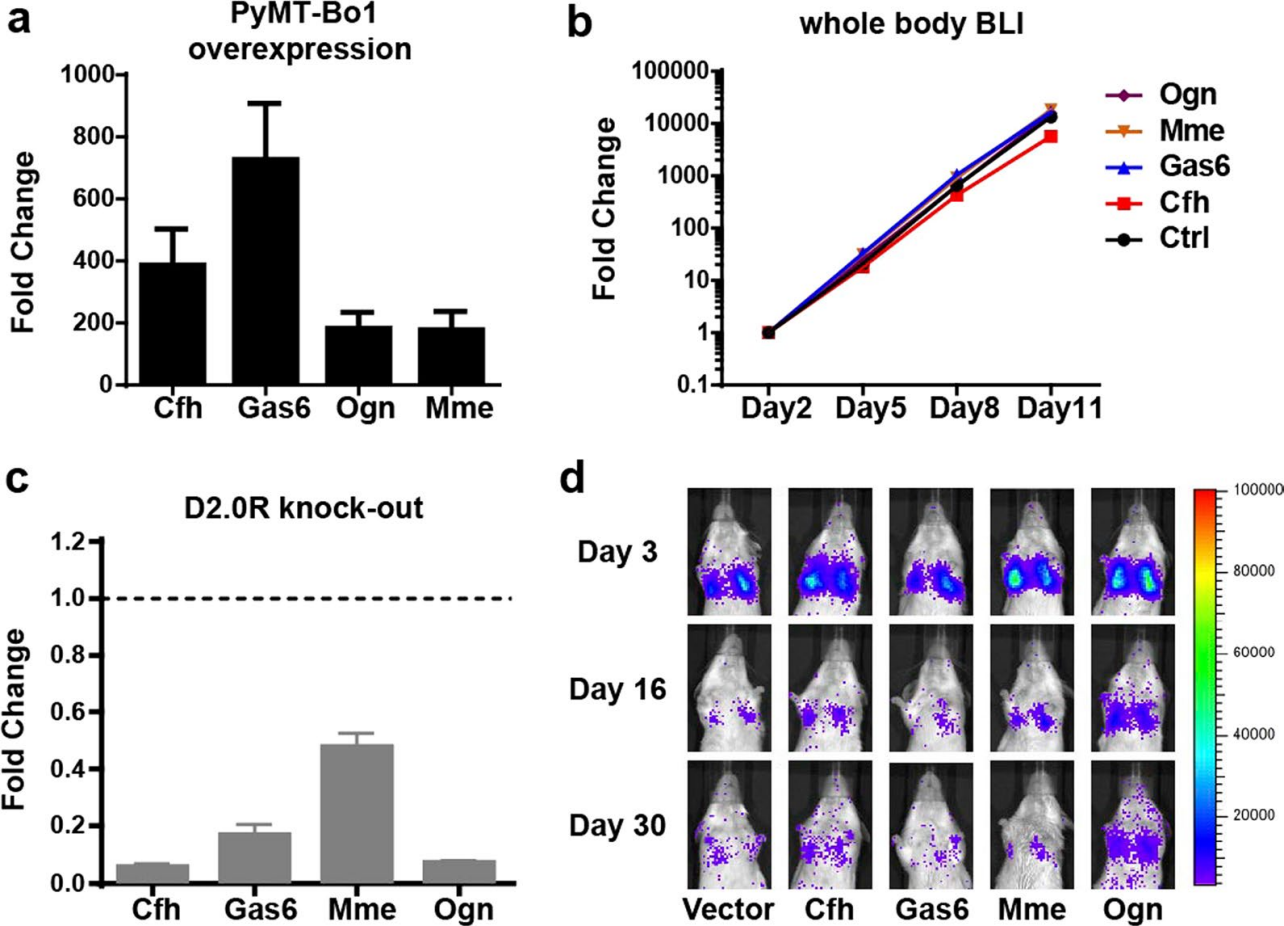
Modulation of dormancy-related genes does not impact the in vivo dormancy phenotype. **a** qRT-PCR analysis of ectopically expressed dormancy genes in PyMT-Bo1 cells (*n* = 3). **b** Whole body BLI tracking of in vivo proliferation of control versus PyMT-Bo1 cells ectopically expressing the genes as indicated, 5 mice per group. **c** Dormancy gene expression in D2.0R cells after CRISPR/Cas9 knockout determined by qRT-PCR, compared to Cas9 expressing D2.0R cells without a gRNA, which was set to 1 (*n* = 3). **d** Representative BLI images tracking the growth of different dormancy gene-KO D2.0R cells in the lung for 30 days, 3 mice per group

Next, we wanted to ask whether the dormancy-related genes (enriched in DiD + cells) that we uncovered in our scRNA-Seq analyses were sufficient to enforce metastatic breast cancer cells to enter dormancy in vivo. Combining our scRNA-seq and qRT-PCR validation results, we selected *Cfh, Gas6, Ogn* and *Mme* as our top candidates because of their high-level expression in DiD + cells. Using lentiviral delivery, we ectopically expressed each gene in PyMT-Bo1 cells (Fig. 6a). We found that introduction of *Cfh, Gas6, Ogn* or *Mme* failed to directly impact cell proliferation in vitro (Additional file 1: Fig. S7a). Next, we introduced *Cfh, Gas6, Ogn* or *Mme* expressing cells via IC injection into albino C57BL6 mice and compared their metastatic capacity. When we compared the growth of *Cfh, Gas6, Ogn* or *Mme* expressing cells to parental PyMT-Bo1 cells, we found no difference in their ability to grow as evidenced by whole body bioluminescence imaging (BLI) (Fig. 6b). In addition, the frequency of DiD + *Cfh*, *Gas6, Ogn* or *Mme* expressing cells also remained the same as parental PyMT-Bo1 cells (Additional file 1: Fig. S7b). Therefore, ectopic expression of individual dormancy-related genes is not sufficient to change a breast cancer cell’s metastatic potential or direct them into a dormant state.

Knowing that both DiD + PyMT-Bo1 cells and dormant D2.0R cells exhibit a similar gene expression signature in vivo and that ectopic expression of *Cfh, Gas6, Ogn* or *Mme* failed to induce a dormant phenotype in PyMT-Bo1 cells, we next asked if those genes were necessary for maintaining breast cancer cell dormancy. To test their necessity, we used the CRISPR/Cas9 system to knockout (KO) individual candidate genes in D2.0R cells. We deleted *Cfh, Gas6, Ogn* and *Mme* from D2.0R cells separately (Fig. 6c). Using this approach, we reasoned that if our genes were sufficient to modulate dormancy, even a small number of reactivated cells would lead to metastatic outgrowth, resulting in increasing BLI intensity over time. After IV injection of *Cfh, Gas6, Ogn* or *Mme* KO D2.0R cells into BALB/c mice, we used BLI to follow cell growth in the lung for over 30 days. Unexpectedly, none of the KO cell lines yielded any metastatic outgrowth in the lung and the tumor signal failed to increase over 30 days (Fig. 6d and Additional file 1: Fig. S7c). Thus, individual manipulation of the dormancy-related genes identified by our scRNA-seq and tested here were neither necessary nor sufficient to regulate breast cancer cell dormancy regardless of where they were seeded. It is possible other genes, not identified in our approach regulate dormancy and/or that it is a multi-gene program rather than an individual gene alone that regulates breast cancer dormancy.

### Dormancy genes predict tumor progression in patients

**Fig. 7:**
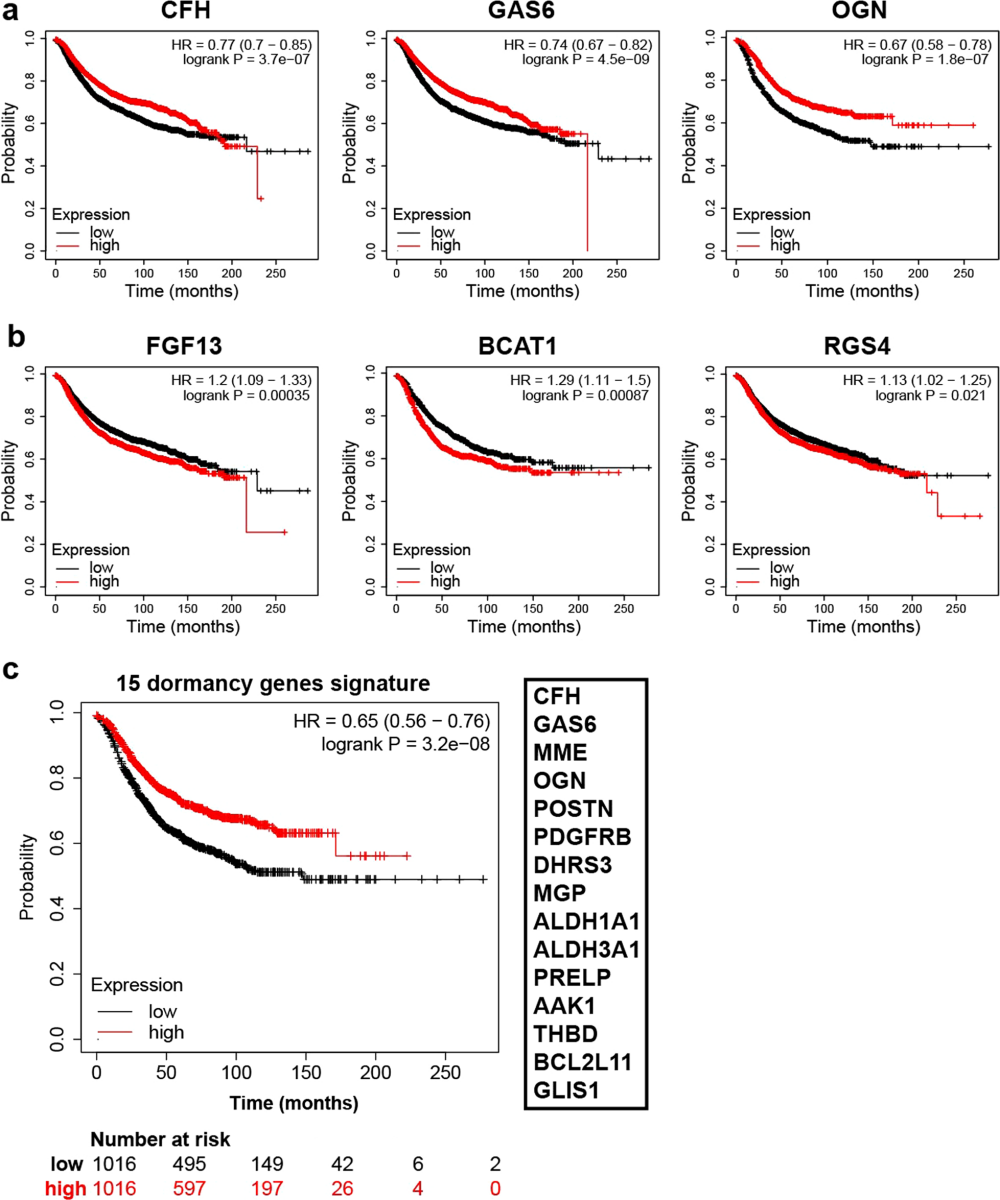
Relapse-free survival analysis of dormancy biomarkers in breast cancer patients. **a**, **b** Kaplan–Meier plots showing breast cancer patients relapse-free survival (RFS) related to genes enriched in dormant (**a**) and proliferative breast cancer cells (**b**). **c** Combining our top-15 enriched genes in dormant breast cancer cells predicts an overall better prognosis among breast cancer patients. All the plots and statistical analyses were generated from Kaplan–Meier Plotter (https://kmplot.com/analysis/)

Our above data indicated that the individual dormancy-related genes that we tested did not control breast cancer dormancy, however, those genes were consistently expressed in dormant breast cancer cells among different models, raising the possibility that their expression might predict which patients are more likely to experience a metastatic recurrence. To address this possibility, we queried whether our genes (i.e., *Cfh, Gas6, Mme, Ogn, Postn, Pdgfrb, Aldh1a1, Dhrs3* and *Mgp*) identified patients less likely to experience a recurrence. Indeed, *Cfh, Gas6* and *Ogn* expression correlated with better relapse-free survival (RFS) among breast cancer patients across several datasets [22] (Fig. 7a). Moreover, patients that harbored tumors expressing higher levels of down-regulated genes in DiD + dormant PyMT-Bo1 cells (including *Fgf13, Bcat1* and *Rgs4* and excluding proliferation genes) exhibited a poorer relapse-free survival (Fig. 7b). Using additional differentially expressed genes in DiD + PyMT-Bo1 cells (Additional file 1: Fig. S8a) plus genes we validated by qRT-PCR (Fig. 4c), we developed a 15-gene signature to identify patients at reduced risk of recurrence. When those 15 genes were combined, there was a clear survival advantage in patients expressing high levels of these genes over those patients expressing lower levels (Fig. 7c), suggesting the disseminated breast cancer cells from primary tumors expressing dormancy-related genes that we identified, are more likely to remain in a dormant state rather than contribute to cancer relapse. Interestingly, when we incorporated genes that were down-regulated in DiD + PyMT-Bo1, we further increased the predictive value of our identified dormancy gene signature (Additional file 1: Fig. S8b).

## Discussion

Dormant DTCs are exceedingly rare and while they have been found in the bones of patients [29], they remain difficult to isolate. This is likely due to both their low numbers and their physical location. Indeed, dormant DTCs in the bone are tightly associate with the endosteal niche [9]. To overcome these challenges, we developed an efficient system that allowed us to robustly isolate rare dormant breast cancer cells from the bones of mice. This approach allowed us to isolate dormant cells from two different breast cancer models from both the lung and the bone and show that there is a common gene expression signature in dormant cells. Interestingly, we find that patients that express this common signature in their primary tumors have an increased overall survival, suggesting this signature can identify those patients that possess tumor cells more likely to remain in a dormant state. We also find that higher expression of CFH, GAS6, ALDH1A, and MGP are also observed in patients more likely to undergo late stage recurrence, suggesting these patients harbored dormant DTCs.

We found dormant breast cancer cells can be identified in multiple metastatic sites after inoculating metastatic breast cancer cells into mice. We successfully isolated dormant PyMT-Bo1 cells from bone and lung where we were surprised to find that cells from both sites expressed common genes including *Cfh, Gas6, Mme, Ogn, Postn, Pdgfrb, Aldh1a1, Dhrs3* and *Mgp*. While we found similarities, we did not carry out scRNA-Seq on cells isolated from the lungs, so there could also be significant differences. The microenvironment is known to play an important role in controlling tumor growth and distinct mechanisms have been identified in the bone and lung [35]. In the lung, alveolar type 1 cells promote the survival of dormant breast cancer cells [10], while a fibrotic environment and neutrophil extracellular traps (NETs) can activate dormant DTCs [13, 14]. In the bone, periarteriolar NG2 + mesenchymal stem cells can induce breast cancer cell dormancy [36], while osteoclast activity can push dormant DTCs into cell cycle [37]. Because PyMT-Bo1 cells induce robust osteoclastogenesis [38], in our system the PyMT-Bo1 may be responsible for initiating remodeling of the dormancy-supporting niche, causing the reactivation of DiD + dormant cells.

Beyond murine models, DTCs have been identified in bone marrow specimens from breast cancer patients. Depending on the method used for DTC identification, 15–60% of early-stage breast cancer patients have DTCs in their bone marrow [29]. Several studies have reported that bone marrow DTCs can predict cancer recurrence and poor survival among breast cancer patients [39–42]. Understanding the genes expressed and the mechanisms active in dormant breast cancer DTCs could be key to preventing cancer recurrence. Interestingly, the dormancy regulator (*Nr2f1*) first identified in a murine prostate cancer model also serves as a biomarker for dormant DTCs in breast cancer patients [17, 30], suggesting that while there are clear differences between the cancer types, there may be common dormancy-related genes. Notably, several studies using different prostate cancer dormancy models have also shown Cfh and Gas6, two of our top dormancy-related genes, are consistently up-regulated in dormant cancer cell populations [7, 16, 17, 43]. Moreover, our data indicate the existence of a gene expression signature that is shared by dormant breast cancer cells residing in different metastatic organs. What remains to be addressed is if the expression of any combination of these genes plays a role in controlling dormancy in the metastatic setting and whether targeting the genes would alter recurrence rates in patients.

We identified a group of genes commonly up-regulated in dormant breast cancer cells, including *Cfh, Gas6, Ogn* and *Mme* in two models of breast cancer dormancy. However, genetic manipulation of individual dormancy-related genes failed to change the metastatic phenotype of breast cancer cells. The fact that dormant D2.0R cells express high levels of those dormancy-related genes but still proliferate to the same extent as metastatic D2A1 in 2D cultures also suggests that those genes do not directly regulate breast cancer cell dormancy or proliferation. However, we did find that patients expressing high levels of these genes have a better prognosis than those who express low levels of the same genes within their primary tumor. Given the difficulty of isolating DTCs from the bones and visceral organs, this finding could help predict which patients are more likely to recur and thus should be subject to more frequent monitoring or additional therapies.

## Conclusion

Our study described an efficient system for the study of rare dormant breast cancer cells in the metastatic sites, including bone and lung. Using this system, we identified a group of genes, including *Cfh, Gas6, Mme* and *Ogn,* that were consistently up-regulated in dormant breast cancer cells compared to proliferative cells. Although genetic manipulation of those genes’ expression in breast cancer cells did not impact their metastatic ability, we found genes enriched in dormant breast cancer cells correlate with recurrence-free survival in breast cancer patients. These data suggest that the genes we identified in dormant breast cancer cells may serve as markers for breast cancer dormancy and prognostic factors for breast cancer patients.

## Supplementary Information

**Additional file 1:** Supplementary Figures S1 to S8.

**Additional file 2:** Supplementary Table S1: List of differentially expressed genes in DiD+ and DiD− PyMT-Bo1 cells isolated from bone.

**Additional file 3:** Supplementary Table S2: List of primer and probe sequences for qPCR analysis of gene expression.

**Additional file 4:** Supplementary Table S3: List of gRNA sequences used to knockout dormancy genes in D2.0R cells.

## Data Availability

The datasets generated and analyzed during the current study have been submitted to Gene Expression Omnibus (GEO), study GSE192802.

## Abbreviations

DTC: Disseminated tumor cell
IC: Intracardiac
BLI: Bioluminescence imaging
scRNA-seq: Single cell RNA-sequencing
FACS: Fluorescence-Activated Cell Sorting
MACS: Magnetic-activated cell sorting
CTFR: CellTrace Far Red
LRC: Label-retaining cell
DGE: Differential gene expression
qRT-PCR: Quantitative real-time reverse transcription PCR
KO: Knockout
RFS: Relapse-free survival
