# Supplementary material for: Gene expression predicts dormant metastatic breast cancer cell phenotype
Source: Breast Cancer Res. 2022 Jan 29;24:10. doi: 10.1186/s13058-022-01503-5 (PMC8800302; doi:10.1186/s13058-022-01503-5)
Supplement: Supplementary file 1 — Additional file 1: Supplementary Figures S1 to S8. [file 13058_2022_1503_MOESM1_ESM.docx]

**
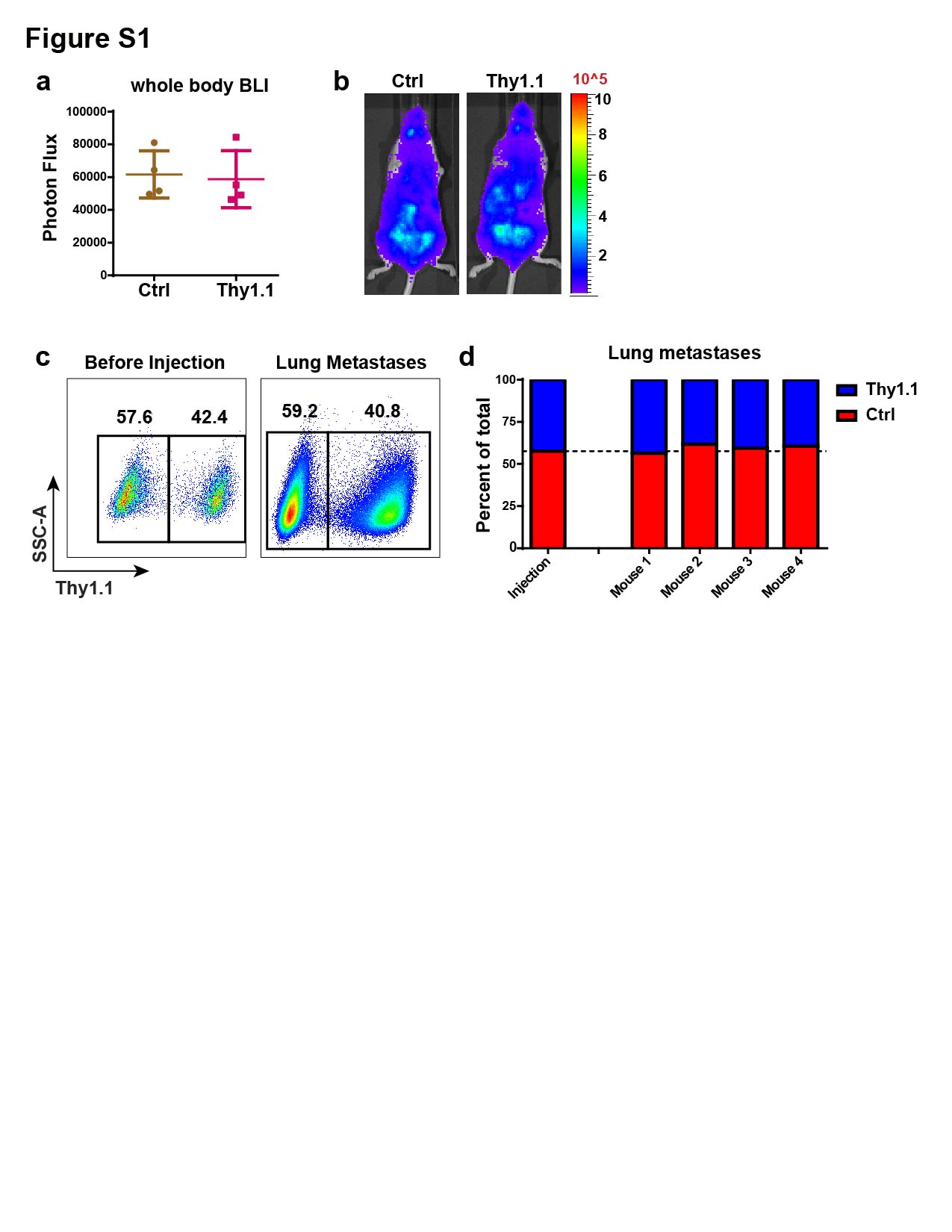

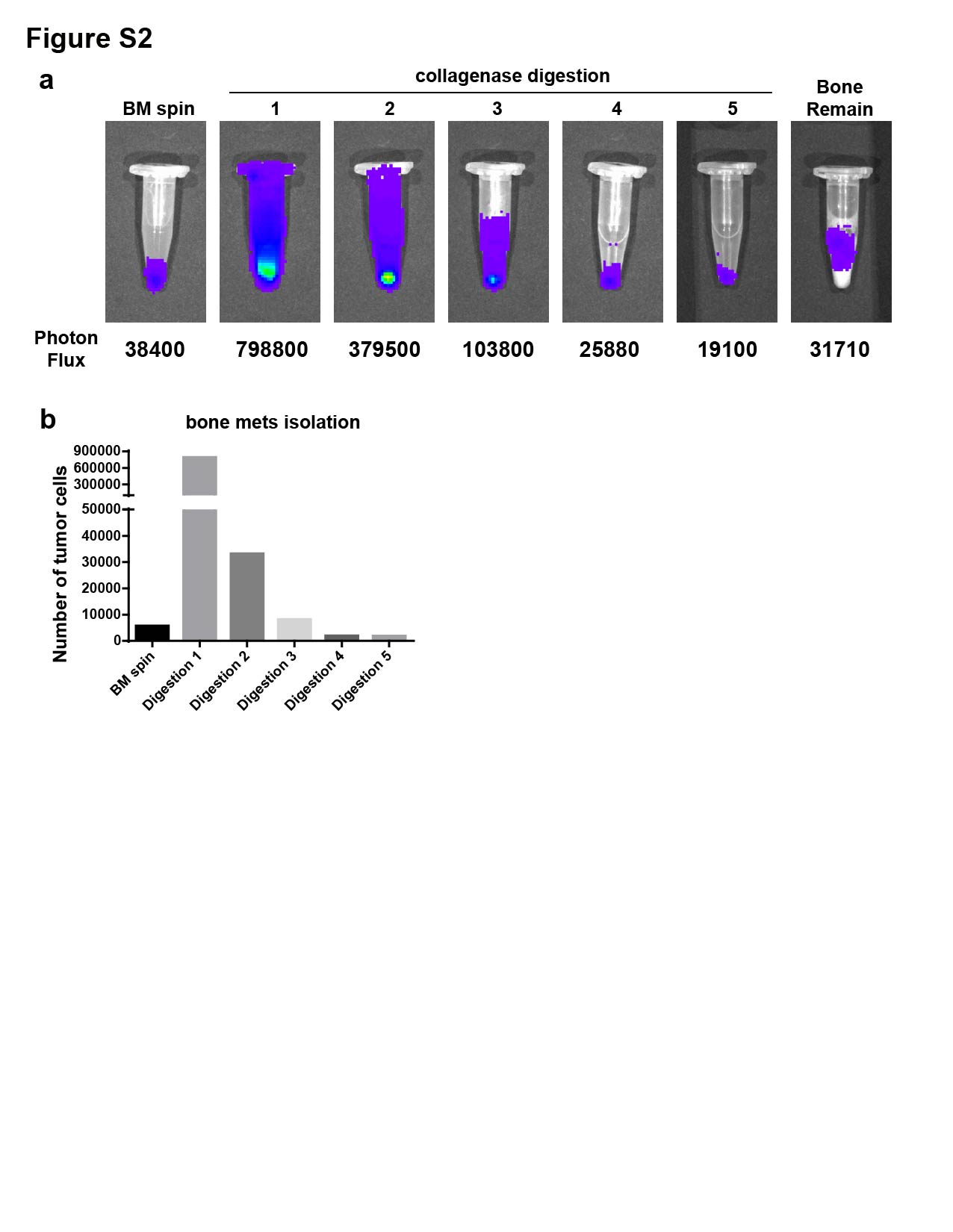

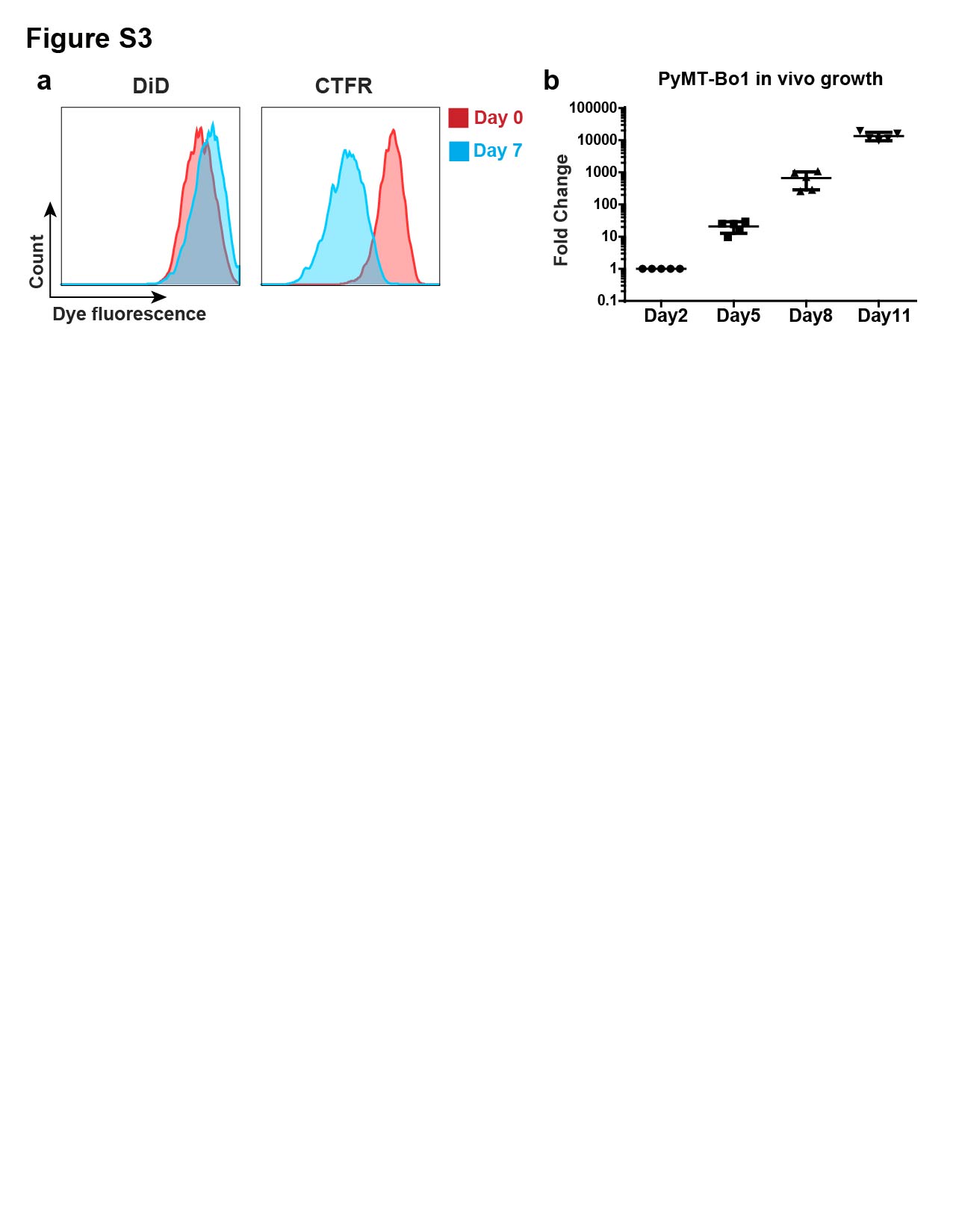

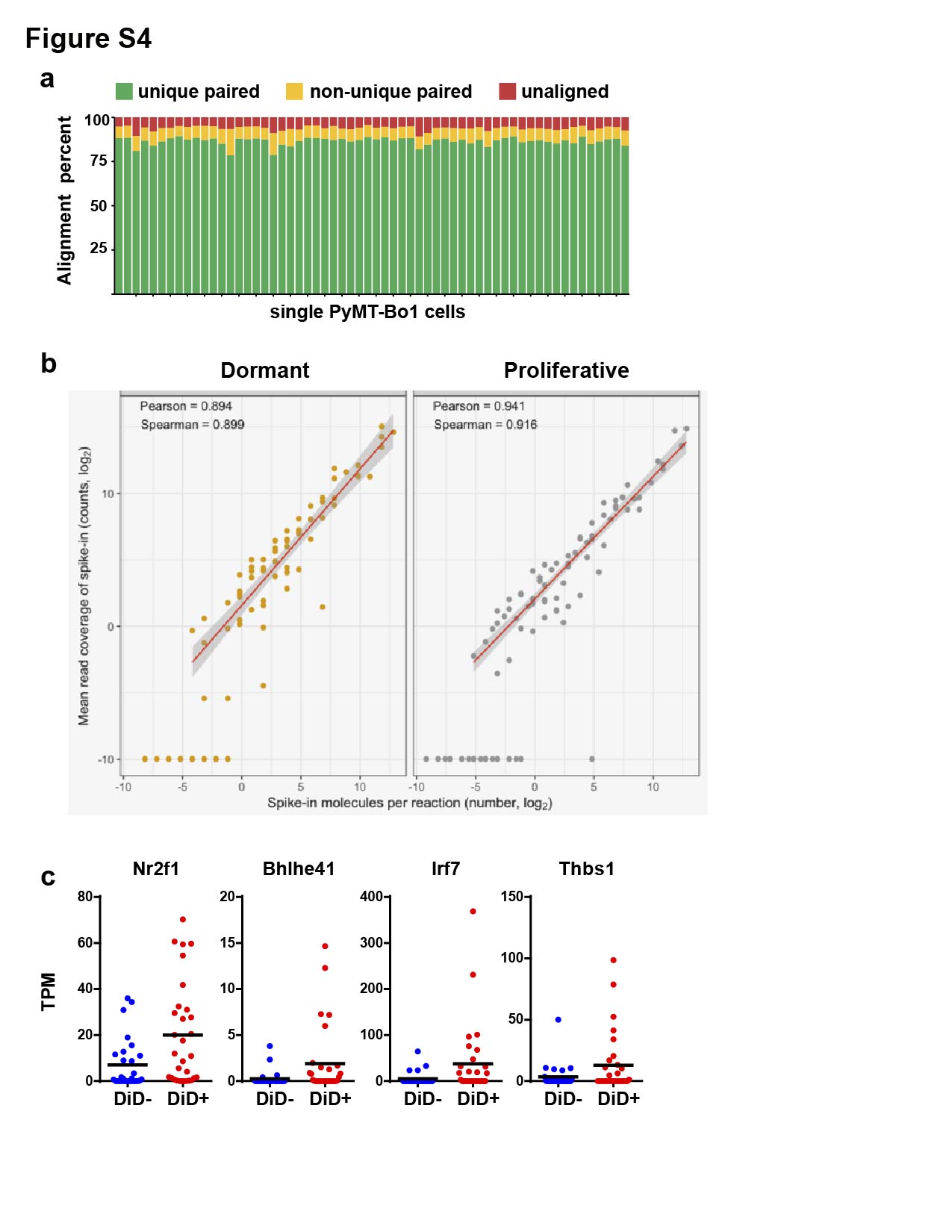

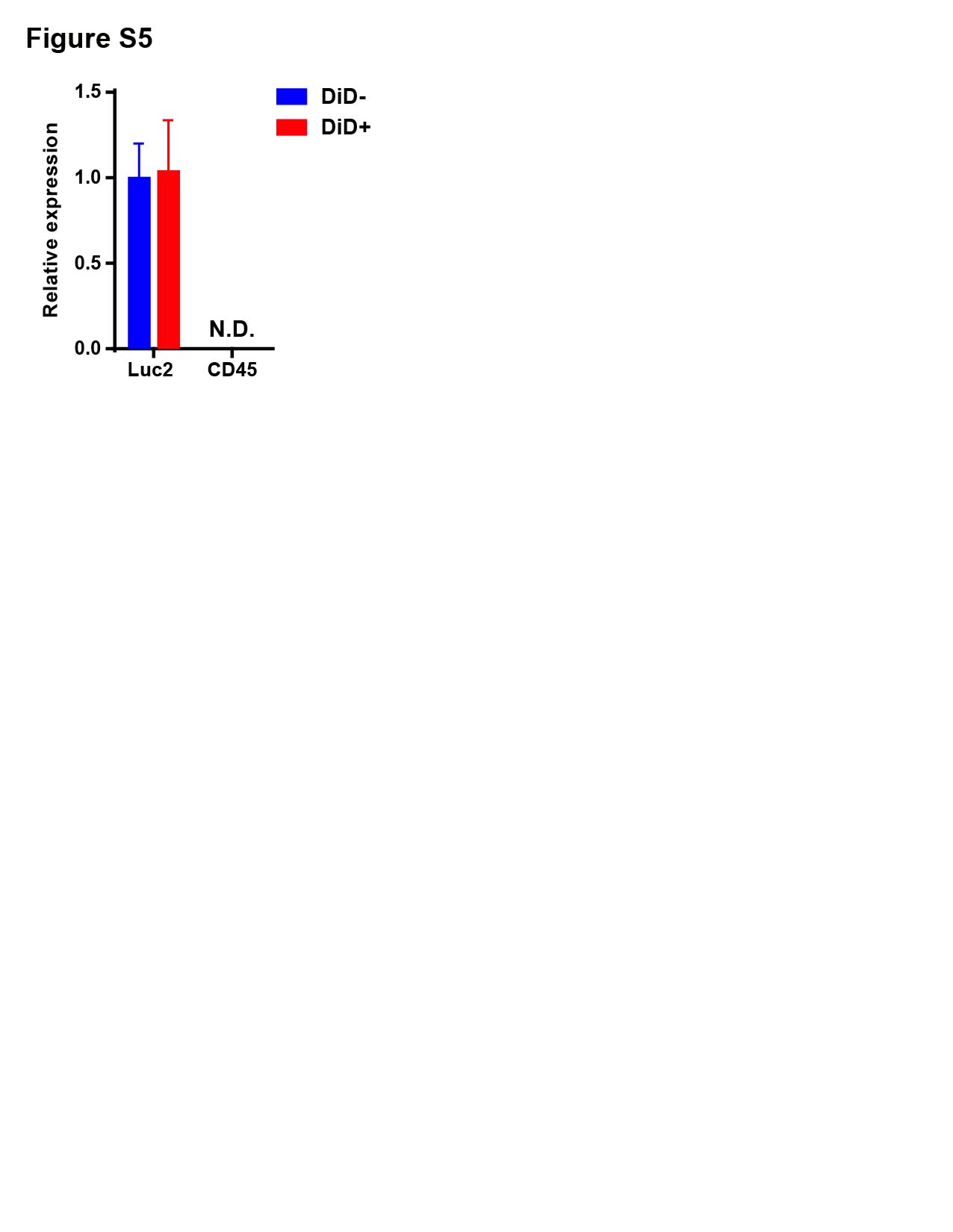

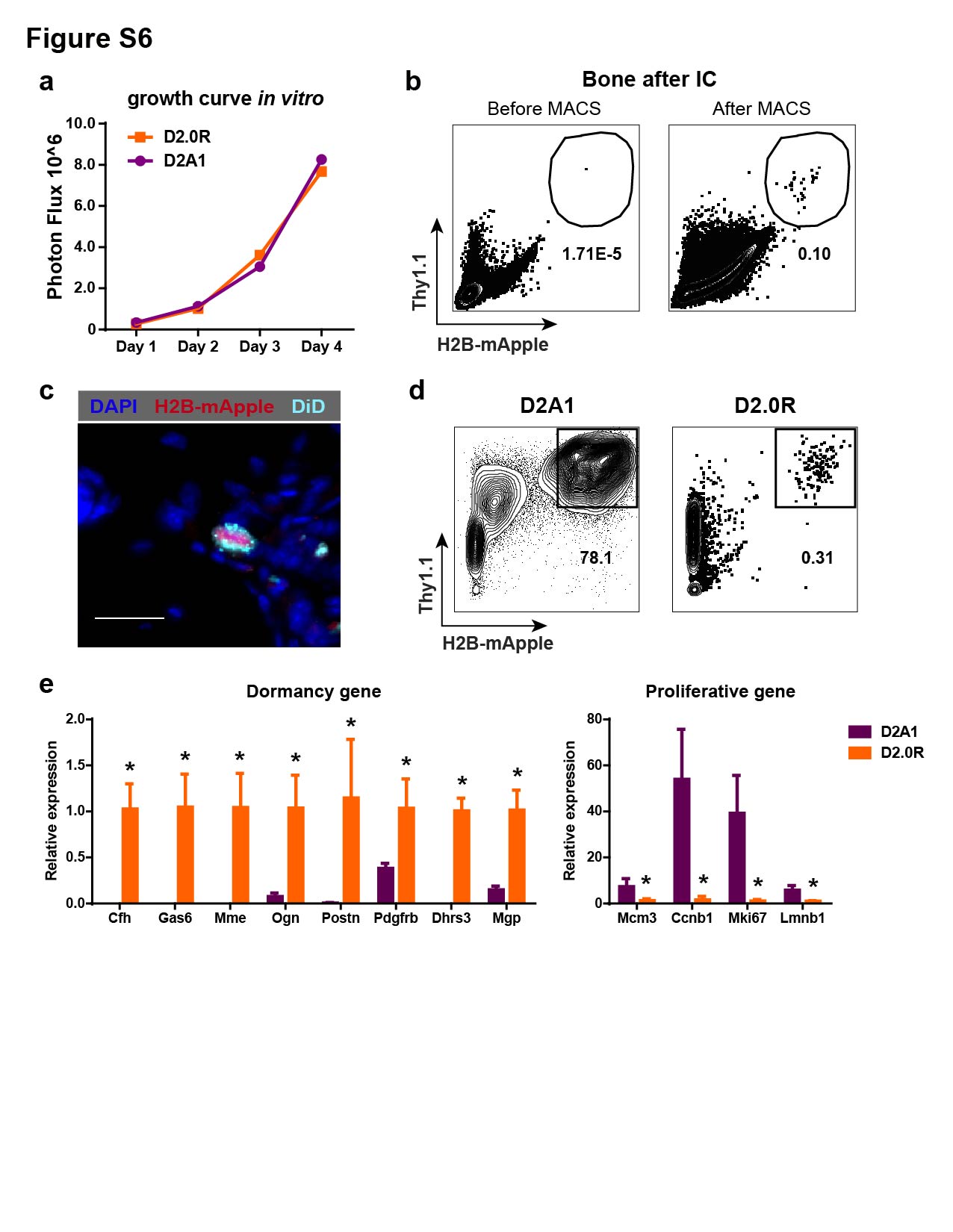

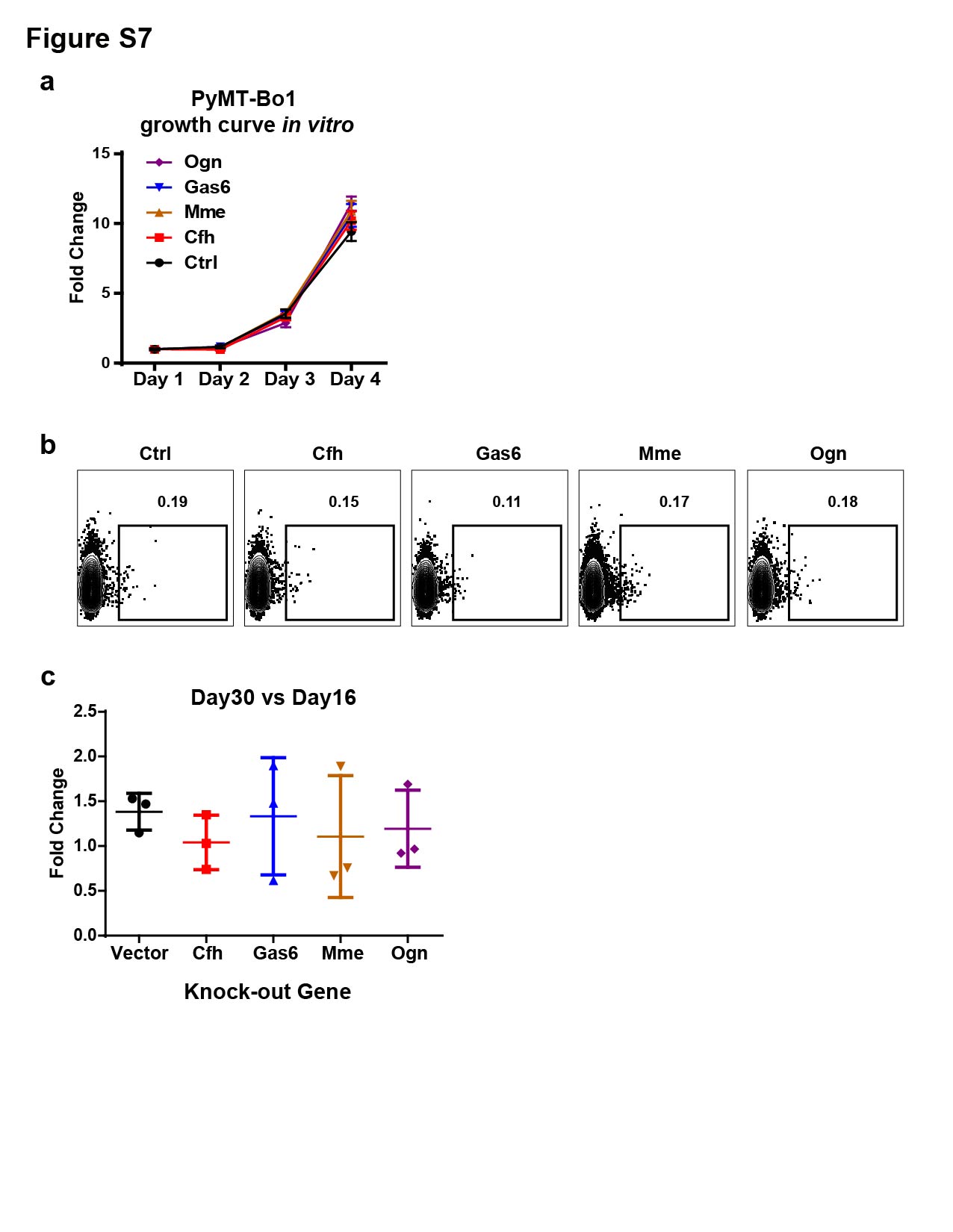

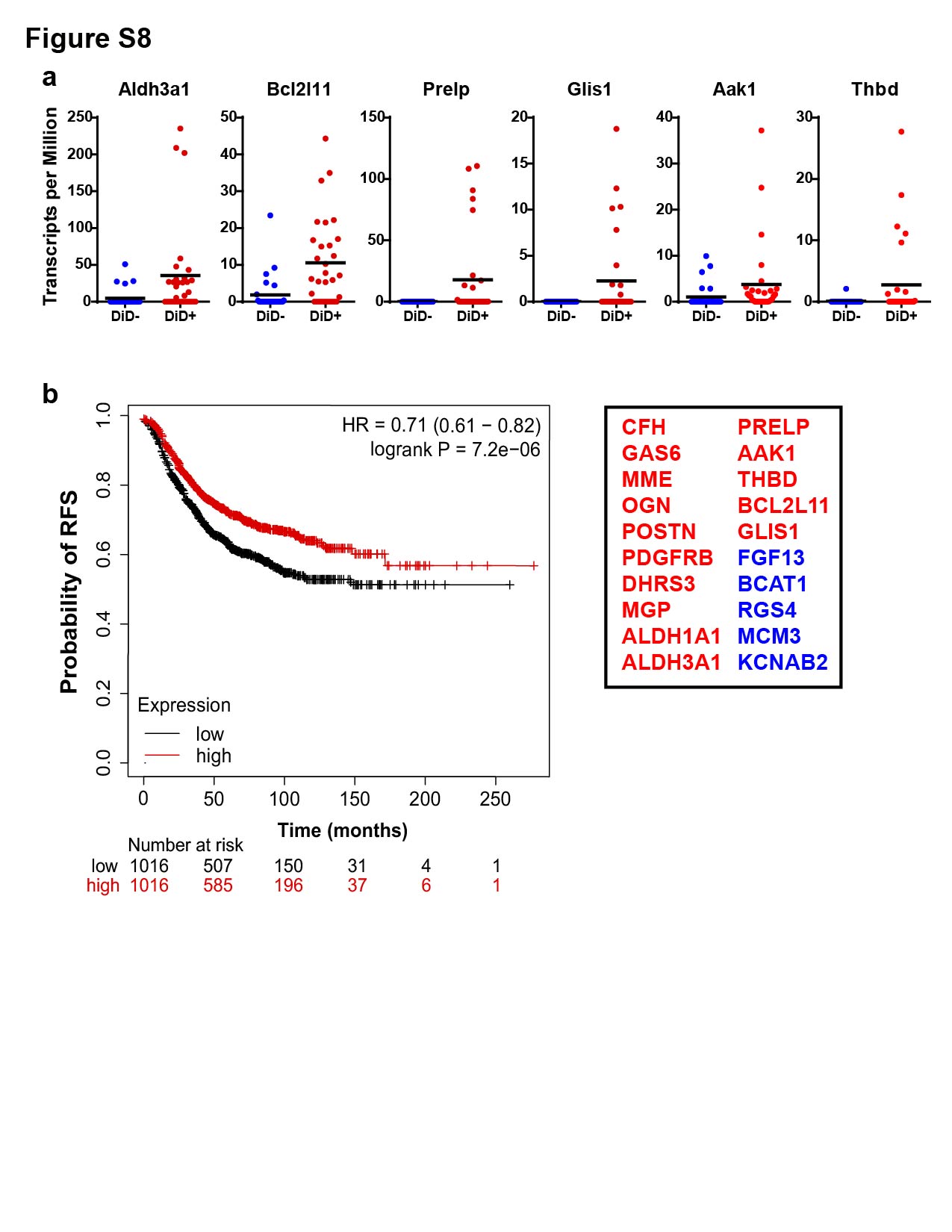
**

**Figure Legends**

**Supplementary Figure S1: Ectopic expression of Thy1.1 in PyMT-Bo1 cells does not impact their metastatic potential. (a)** BLI comparing Control vs Thy1.1+ PyMT-Bo1 metastatic growth *in vivo* 7 days after IC injection. **(b)** Representative image of Control vs Thy1.1+ PyMT-Bo1 signals 7 days after IC injection. Scale represents photon flux (photons/sec/cm^2^/sr). **(c)** Flow cytometry analysis of mixed control and Thy1.1+ PyMT-Bo1 cells before and after IV injection into mice. **(d)** Summary of competitive growth comparison between control and Thy1.1+ PyMT-Bo1 cells *in vivo.* n=4.

**Supplementary Figure S2: PyMT-Bo1 isolation from bones after serial enzymatic digestion. (a)** BLI image showing PyMT-Bo1 cells isolated from tumor-bearing bones after multiple rounds of collagenase digestion. **(b)** Summary of flow cytometry quantitation of recovered PyMT-Bo1 cells from each round of collagenase digestion.

**Supplementary Figure S3: DiD membrane dye intensity reflects cellular division. (a)** Flow cytometry comparing DiD and CellTrace Far Red (CTFR) dye retention over 7 days in arrested irradiated mouse embryonic fibroblasts. **(b)** Whole body BLI imaging of tri-labelled PyMT-Bo1 cells *in vivo* growth after IC injection (n = 5).

**Supplementary Figure S4: scRNA-seq quality control. (a)** Summary of STAR mapping results for each single sorted cell. **(b)** Comparing ERCC spike-ins controls in dormant and proliferative PyMT-Bo1 cells. **(c)** Transcript profile for additional previously-reported dormancy genes in dormant (DiD-) versus proliferative (DiD+) cells.

**Supplementary Figure S5: Isolated DiD high cells are free of contaminating CD45 positive cells.** qPCR comparing transgene Luc2 and CD45 expression in sorted PyMT-Bo1 cells from bone (n = 4). N.D, not detected.

**Supplementary Figure S6: Isolation of breast cancer cells from bone. (a)** D2A1 and D2.0R cells were seed at 500 cells per well in 96-well plate. Growth was measured by BLI every day for 4 days (n = 8). **(b)** Flow cytometry detection of D2.0R DTCs isolated from the bone before and after MACS enrichment. **(c)** Fluorescence image of single DiD+ D2.0R DTC residing in the bone after IT injection where no tumor growth was observed by BLI (data not shown). Scale bar = 50 um. **(d)** FACS sorting of D2A1 and D2.0R cells from the bone after IT injection. **(e)** qPCR results comparing dormancy-related and proliferation genes in sorted D2A1 (n = 4) and D2.0R cells (n = 3). Significance was determined by unpaired t tests, *p≤0.05.

**Supplementary Figure S7: Genetic manipulation of dormancy gene expression in PyMT-Bo1 and D2.0R cells. (a)** BLI to detect PyMT-Bo1 cell growth following ectopic expression of dormancy genes (n = 9). Cells were seeded at 500 cells per well in a 96-well plate and cultured for 4 days with daily BLI measurements. **(b)** FACS analysis of DiD+ label-retaining PyMT-Bo1 cells ectopically expressing dormancy genes from bone 11 days after IC injection. **(c)** BLI of lung region from mice after IV injection of D2.0R cells. Shown is BLI of D2.0R cells on Day 30 vs Day 16, showing no change.

**Supplementary Figure S8: Expression of additional dormancy genes identified by scRNA-seq. (a)** Transcripts profiles for additional top differentially expressed genes in dormant DiD+ and proliferating DiD- PyMT-Bo1 cells included in 15-gene dormancy signature according to our scRNA-seq. **(b)** Kaplan-Meier Plots showing 20-gene signature (combining our top-15 enriched genes and top-5 down-regulated genes (inverted) in dormant breast cancer cells) predicts further increased RFS among breast cancer patients, compared to the gene signature presented in **Fig. 7c**.
